# Design, Optimization and Performance Assessment of Single Port Film Bulk Acoustic Resonator through Finite Element Simulation

**DOI:** 10.3390/s23218920

**Published:** 2023-11-02

**Authors:** Raju Patel, Manoj Singh Adhikari, Shailendra Kumar Tripathi, Sourabh Sahu

**Affiliations:** 1 School of Electronics Engineering (SENSE), Vellore Institute of Technology (VIT), Chennai 600127, India; raju.patel@vit.ac.in; 2 School of Electronics & Electrical Engineering, Lovely Professional University, Phagwara 144411, India; manoj.space99@gmail.com; 3 Department of Electronics & Communication Engineering, KL University, Guntur 522502, India; shailendra.amu@gmail.com; 4 Department of Electronics & Communication Engineering, Gyan Ganga Institute of Technology and Sciences, Jabalpur 482003, India

**Keywords:** MEMS, single port acoustic resonator, zinc oxide, structural morphologies, vector network analyzer

## Abstract

In this paper, the study is supported by design, FEA simulation, and practical RF measurements on fabricated single-port-cavity-based acoustic resonator for gas sensing applications. In the FEA simulation, frequency domain analysis was performed to enhance the performance of the acoustic resonator. The structural and surface morphologies of the deposited ZnO as a piezoelectric layer have been studied using XRD and AFM. The XRD pattern of deposited bulk ZnO film indicates the perfect single crystalline nature of the film with dominant phase (002) at 2θ = 34.58°. The AFM micrograph indicates that deposited piezoelectric film has a very smooth surface and small grain size. In the fabrication process, use of bulk micro machined oxide (SiO_2_) for the production of a thin membrane as a support layer is adopted. A vector network analyzer (Model MS2028C, Anritsu) was used to measure the radio frequency response of the resonators from 1 GHz to 2.5 GHz. As a result, we have successfully fabricated an acoustic resonator operating at 1.84 GHz with a quality factor Q of 214 and an effective electromechanical coupling coefficient of 10.57%.

## 1. Introduction

Modern mobile communication systems have become increasingly pervasive, driving the need for efficient power utilization, spectrum optimization, and high-quality performance. However, the existing integrated circuit (IC) technology has encountered limitations in reducing the size of RF components required for these systems. Despite this, advancements in micro-fabrication processes have facilitated the production of these components, even within the constraints of current silicon CMOS technology [1,2,3,4,5,6]. Surface acoustic wave (SAW) and quartz crystal microbalance (QCM) resonators are capable of operating in the frequency range of up to 500 MHz. In contrast, microwave ceramic resonators can operate in the GHz range but suffer from bulkiness. These resonators are typically integrated as discrete devices with high insertion losses on the silicon IC, resulting in a larger system area and subsequent deterioration in temperature sensitivity and power handling [7,8].

To address these challenges, researchers have been exploring innovative solutions to enhance the performance and integration of RF components within mobile communication systems. By leveraging novel fabrication techniques and materials, it becomes possible to develop miniaturized and efficient components that can meet the demands of modern wireless applications. One such approach involves the utilization of nanoscale materials, such as carbon nanotubes (CNTs) and graphene, which exhibit exceptional electrical properties and mechanical strength. These materials have shown promise in enabling the development of compact and high-performance RF components. For instance, CNT-based resonators have demonstrated superior frequency stability and reduced power consumption compared to conventional technologies [9,10,11,12]. Another avenue of research focuses on the integration of RF components using advanced packaging techniques. System-on-chip (SoC) technology, which combines multiple functionalities on a single chip, offers a potential solution for reducing the system area and mitigating insertion losses. Additionally, three-dimensional (3D) integration techniques provide a means of vertically stacking multiple layers of RF components, thereby further reducing the overall system footprint [13,14,15].

Furthermore, the emergence of new materials, such as piezoelectric polymers and ferroelectric thin films, holds promise for the development of compact and high-performance resonators. These materials exhibit excellent electromechanical properties and can be integrated directly into silicon substrates, enabling the monolithic integration of RF components [16,17,18,19]. Researchers have made significant advancements in the fabrication of Film Bulk Acoustic Resonators (FBARs) using Microelectromechanical Systems (MEMS) technology and compatible Piezoelectric (PZE) materials. Several PZE materials, including Zinc Oxide (ZnO), Aluminium Nitride (AlN), Gallium Nitride (GaN), magnesium-doped zinc oxide (MgxZn1-xO), and PZT, have been successfully employed in the fabrication of FBARs [20,21,22,23,24]. Additionally, researchers have explored the use of ferroelectric materials such as barium strontium titanate (BaxSr1-xTiO_3_, BST), strontium titanate (SrTiO_3_, STO), and barium titanate (BaTiO_3_, BTO) for FBAR fabrication. Among the various PZE materials investigated, AlN and ZnO have emerged as promising candidates for thin film FBAR fabrication due to their desirable properties. In particular, zinc oxide has shown significant potential as a PZE material for FBARs, primarily attributed to its high coupling coefficient within the 1–2 GHz frequency range [25,26,27,28].

The choice of zinc oxide as a PZE material for FBAR fabrication is based on several advantageous characteristics that it possesses. Firstly, its high coupling coefficient ensures efficient energy transfer between electrical and mechanical domains, leading to enhanced resonator performance. Additionally, zinc oxide exhibits excellent piezoelectric properties, including a high electromechanical coupling factor, low mechanical losses, and a stable frequency response. These attributes are crucial for achieving accurate frequency control and high Q-factor in FBAR devices. Zinc oxide thin films can be readily deposited using various techniques such as sputtering, chemical vapor deposition (CVD), and atomic layer deposition (ALD). This versatility in deposition methods enables the fabrication of FBARs on a wide range of substrates, including silicon, glass, and flexible materials [29,30,31,32].

In recent years, environmental pollution problems have increased extensively due to encroachment on technology. All living organisms are exposed to tremendous risks as a result of atmospheric pollution; hence, it is imperative the pollution level of gases, and, consequently, their effect on change in the climate, be monitored. MEMS-based gas sensor is one of the most promising applications currently available. Until now, several attempts have been made for gas sensing using acoustic wave resonators, mostly with quartz crystal microbalance (QCM), cantilever, and surface acoustic wave (SAW) devices with remarkable results. Since the sensitivity of acoustic sensors increases with the square of the resonant frequency, high-frequency acoustic resonators are preferred. QCM sensors are well-established gravimetric sensors which have been in use since 1972. However, their LOD lies in the order of a few nanograms, rendering it inadequate for the detection of low concentrations (<100 nM). To lower the LOD to the pictogram and femtogram range, higher resonant frequency sensors are needed, a phenomenon which have boosted the development of SAW and FBAR resonators based on PZE thin films operating in the GHz range. SAW-based resonators have a range of up to 1 GHz due to the pitch spacing limitation of surface interdigitated electrodes (IDT). By contrast, the FBAR-based resonators are made for high-frequency and have a range of up to a few GHz. The FBAR-based sensor shows a much higher sensitivity in the field of gravimetric sensors, as well as in biological and chemical sensing and has contributed to substantial progress in applications of gas sensing [33,34,35,36,37,38,39,40,41].

One of the main issues with FBAR technologies is the propagation of lateral (Lamb) waves, which result in the creation of undesirable spurious (transverse) modes and higher harmonic modes. These stimulated spurious modes have the ability to produce new lateral modes in true FBAR devices because of the discontinuous edges and bounds. Due to energy conversion into these erroneous modes, these transverse modes can reduce the $k_{eff}^{2}$ and quality factor as well as generate disruptive and undesirable ripples in the filter’s pass band. FBARs are of great interest for sensor and RF filter applications owing to their superior performance characteristics as compared to QCM and SAW resonator. Researchers in this field earlier developed the frame-like FBARs to remove spurious modes in order to attain superior qualities. Unwanted higher harmonic modes are another factor that affects how well FBAR functions but have not been well researched because there is inadequate theoretical understanding of the structure [42,43,44,45].

In our previous work the performance parameter of the FBAR is proposed using an active area optimization technique [4]. With the incorporation of the active area the spurious modes and higher order harmonic modes are suppressed with the confinement of the acoustic signals at the central part due to the tuning of the resonance frequency. This methodology is comparatively easier to fabricate with less complications. 

In this article, we performed the 2D FEA simulation for computing the thickness of oxide (SiO_2_), bottom electrode, piezoelectric layer and top electrode to design the FBAR with 1.76 GHz frequency. 2D FEA simulation permits to perform the frequency domain analysis for finding the different resonance modes. The main objective of this analysis is to obtain the resonant frequency, anti-resonant frequency, quality factor, and coupling coefficient of the designed resonator. Further, active area optimization technique has been used for obtaining the perfect confinement of acoustic signal by suppressing the spurious modes and higher harmonic modes. Optimize design parameter was used to design the mask of the resonator using L-edit Pro v13.00 software. In the fabrication process, the first bulk piezoelectric (ZnO) layer deposition method was optimized using RF reactive magnetron sputtering on the bottom aluminum electrode for the (002) plane. The morphology of the deposited piezoelectric film is inspected using XRD, FESEM, and AFM. The optimized device with an active area of 310 × 310 µm^2^ and a backside air cavity was then fabricated using a TMAH setup. It was finally characterized for its RF measurement using a vector network analyzer. Frequency response of fabricated FBAR were analyzed in detail with respect to 2D FEA simulation. 

## 2. Design and FEA Simulation of Single-Port Cavity-Based FBAR

In this section, a comprehensive study of FBAR performance characteristic of a cavity-based structure is delineated using COMSOL Multiphysics 4.4 FEM tool. The interaction between the electrical potential and displacement is represented by the following piezoelectric constitutive equations [1].

(1) $$T_{i}=c_{ij}^{E}S_{j}-e_{ij}E_{j}$$

where Ti, cij, Sj, eij, and Ej are the stress, stiffness constant, strain, and piezoelectric stress components, respectively.

(2) $$D_{i}=\varepsilon_{ij}^{S}E_{j}+e_{ij}S_{j}$$

where Di is the electric displacement and εij is the permittivity constant. Further, the superscript E is calculated at a constant electric field and the superscript S is calculated at a constant strain.

**Figure 1:**
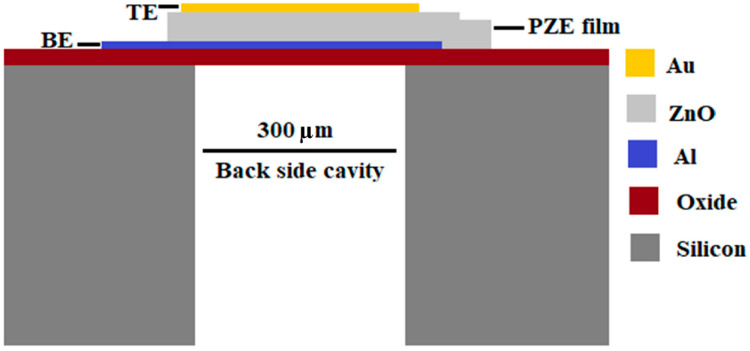
2D schematic of the single-port cavity-based FBAR.

In back-trench-(cavity)-based FBAR, the cavity is formed by etching substrate under the resonating structure, as shown in Figure 1. In this configuration, the acoustic wave is reflected due to a mismatch in acoustic impedance when the bottom Al electrode is exposed to the air through the backside cavity, which is considered to be insignificant acoustic impedance.

The proposed design of cavity-based FBAR is shown in Figure 1. An SiO_2_ layer, as an electrical insulator, is deposited on the Si substrate (0.9 µm thick) and lies between the Si substrate and the bottom electrode. The resonating structure consists of a PZE layer made of zinc oxide (ZnO) that is sandwiched between two metal electrodes and is mounted on the SiO_2_ layer. For this design, a 1.5 µm thick PZE layer was chosen, with 0.15 µm thick electrodes.

The 2D FEM geometry of cavity-based FBAR consists of the following layers: a silicon substrate, SiO_2_, a bottom Al electrode, ZnO, and an Au electrode at the top. In this geometry, the top Au electrode consists of intermittent layers with a free verge. A fixed (ux = uz = 0) border constraint was used at the right and left sides of the geometry.

**Figure 2:**
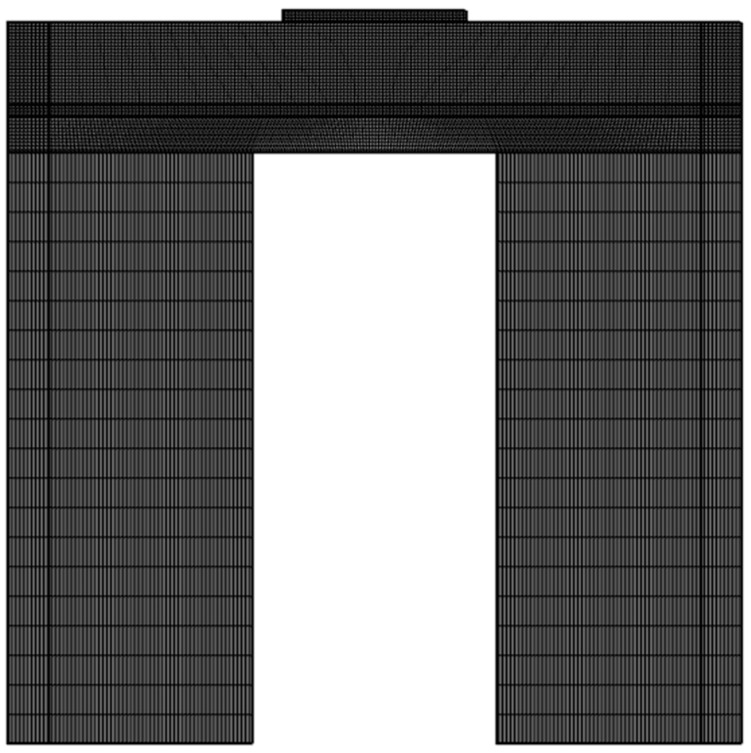
Two-dimensional mapped meshing structure of FBAR.

To simulate the effect of absorption and propagation of the elastic wave in the adjacent region, the lateral dimensions of the resonator are increased by adding a 50 µm wide perfectly matched layer (PML) on both sides of all the layers except for the top electrode. A two-dimensional FEA simulation allows for the frequency domain analysis to be performed to identify the different resonance modes. The selection of different materials for the resonator with thickness, PML layer, and fixed boundary conditions is performed in this step. Further, mapped meshing was utilized for the finite element COMSOL simulation of the resonator. The 2D mapped meshing structure of FBAR is illustrated in Figure 2. The main objective of this analysis is to obtain the resonant frequency, anti-resonant frequency, quality factor, and coupling coefficient for the designed resonator.

When a voltage is applied to the piezoelectric layer, the centrosymmetry of the piezoelectric crystal structure breaks and the effect of converse piezoelectricity is induced. The generated mechanical wave results in a mechanically induced polarization due to the direct piezoelectric effect. When such a mechanically induced polarization is out of phase with the dielectric polarization by 180°, parallel resonance and net polarization, and hence the net current, occur, with both being minimized. The displacement profile, phase, impedance, and quality factor responses of the cavity-based FBAR were illustrated using frequency-domain finite element analysis. The frequencies corresponding to the minimum and maximum values of the electrical impedance Z were considered as resonant frequency fr and anti-resonant frequency fa, respectively. To evaluate the FBAR performance, the value of the quality factor, Q, at resonant and anti-resonant frequencies, the effective electromechanical coupling coefficient, $k_{eff}^{2}$, and the figure of merit (FoM) are deduced from the standard equations [1,2]:

(3) $$Q_{f_{x}}=\frac{f_{x}}{2}{\left|\frac{d\phi_{z}}{df}\right|}_{f_{x}}$$

(4) $$k_{eff}^{2}=\frac{\left(\frac{\pi }{2}\right)\left(\frac{f_{r}}{f_{a}}\right)}{tan\left(\left(\frac{\pi }{2}\right)\left(\frac{f_{r}}{f_{a}}\right)\right)}\;\approx \;{\left(\frac{\pi }{2}\right)}^{2}\frac{f_{a}-f_{r}}{f_{a}}$$

(5) $${FoM}_{1}=k_{eff}^{2}*Q$$

(6) $${FoM}_{2}=f_{a}*Q$$

where ***ϕ***z is the phase of the electrical impedance, Z, f_x_ is the resonant frequency, f_r_ is the anti-resonant frequency, and f_a_ is the acoustic resonator [1,2].

**Figure 3:**
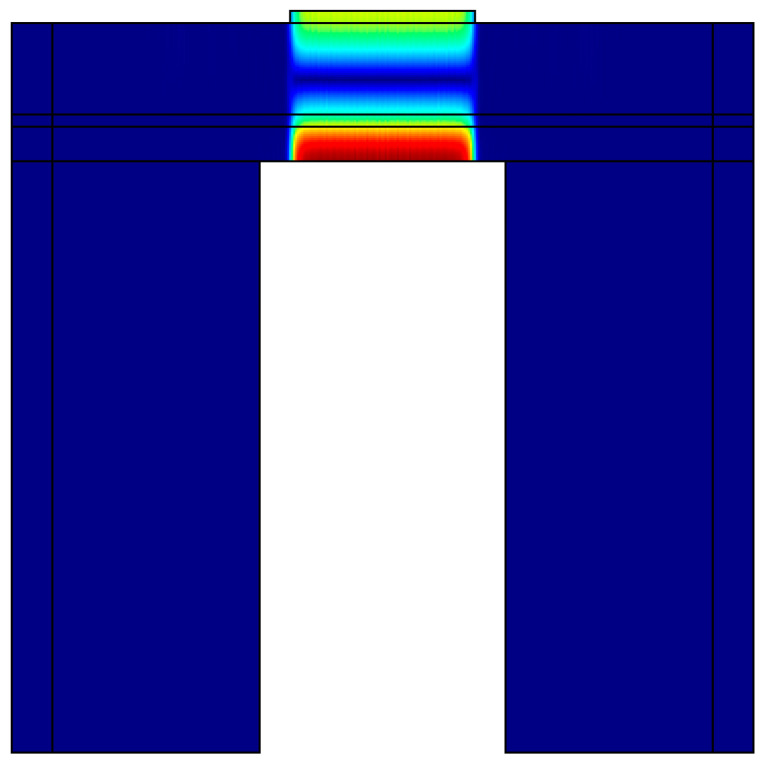
Displacement profile of the cavity-based single-port FBAR, at resonant frequency.

The FEM plot depicted in Figure 3 provides insights into the longitudinal displacement at the resonant frequency. The maximum displacement of 2.02 nm was achieved at the resonant frequency due to the longitudinal mode displacement of the acoustic signal in the active region of the FBAR. Conversely, the remaining section of the FBAR had a minimum displacement of 0 nm.

**Figure 4:**
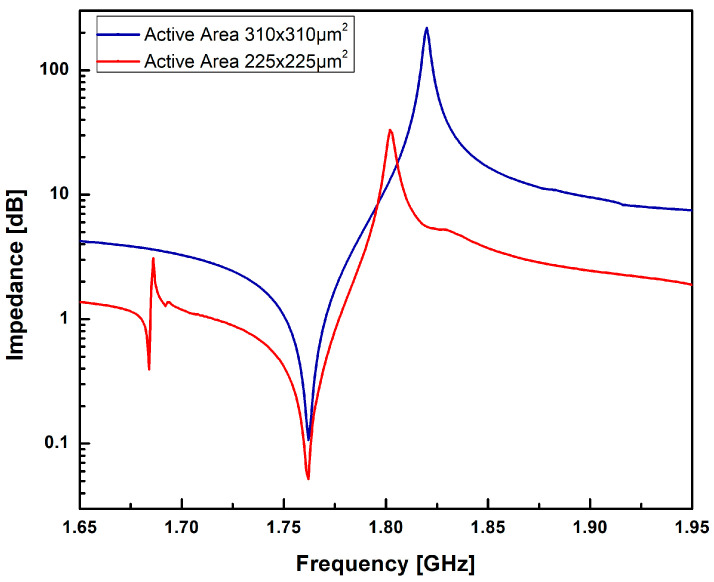
Impedance vs. frequency response of the single port cavity based FBAR for different active area.

**Figure 5:**
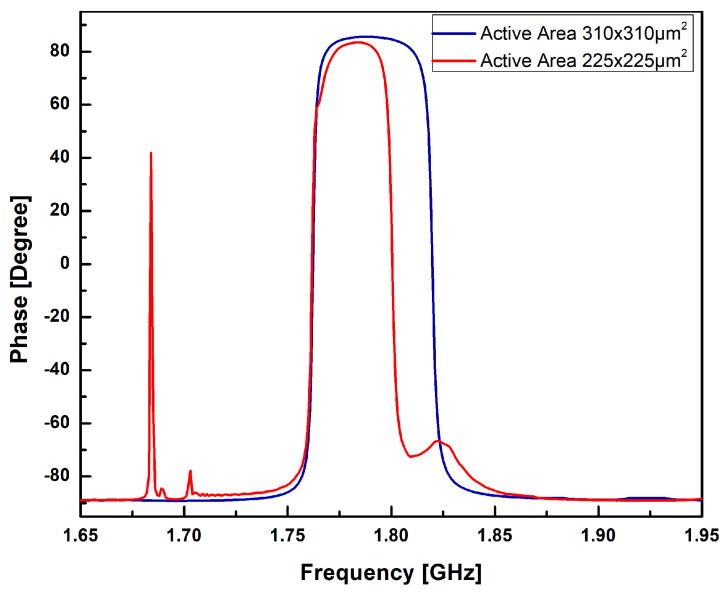
Phase vs. frequency response of the single port cavity based FBAR for different active area.

The displacement profile demonstrates the effective confinement of the acoustic signal within the piezoelectric (PZE) layer, with minimal dissipation of acoustic energy into the silicon (Si) substrate. This confinement is crucial for achieving efficient resonator performance and minimizing signal losses. Examining Figure 4 and Figure 5, the variation of impedance and phase with frequency is illustrated for different active area. It confirms the confinement of acoustic signal by utilizing the active area optimizing techniques. The frequency response from the results also demonstrates that the optimisation technique is suitable to suppress higher harmonic and spurious modes. The resonant frequency, denoted as fr, is determined to be 1.762 GHz, accompanied by a least impedance of 0.11 dB. This resonant frequency signifies the point at which the FBAR exhibits maximum sensitivity to the input signal, resulting in optimal performance. On the other hand, the anti-resonant frequency, de-noted as fa, is measured to be 1.82 GHz, correlating with a maximum impedance of 219.32 dB. The anti-resonant frequency represents the point at which the FBAR demonstrates minimum sensitivity to the input signal.

The effective electro-mechanical coupling coefficient $k_{eff}^{2}$ has been calculated 7.86%. The quality factors at resonant frequency, Qr, and anti-resonant frequency, Qa, are 906.6 and 612.1 with corresponding Figure of Merit’s (FoM1) of 71.26 and 48.11, respectively.

The significant difference in impedance values between the resonant and anti-resonant frequencies highlights the distinctive electrical response of the FBAR device. This disparity plays a crucial role in various applications such as signal filtering and frequency modulation. The observed impedance characteristics and the distinct resonant and anti-resonant frequencies validate the effective operation of the FBAR device. The impedance values obtained provide important information for the optimization of the device performance and the design of appropriate matching circuits.

## 3. Device Fabrication

**Figure 6:**
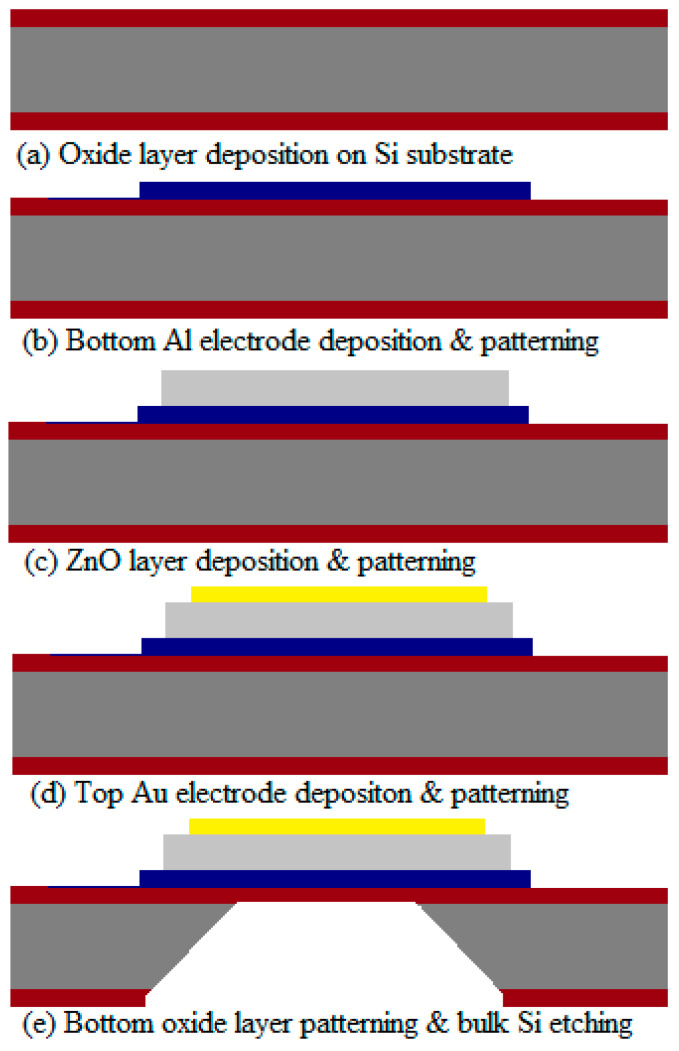
The essential steps in the device fabrication process flow.

The 2″ double side polished Si (100) wafer and four photo masks with the required specifications were used for the fabrication of optimized single-port FBAR. The essential steps in the device fabrication process flow are presented in Figure 6.

The fabrication process of the device began with a series of cleaning steps to ensure the removal of organic residues and contaminants. Degreasing was carried out initially, followed by a standard piranha cleaning process (H_2_SO_4_:H_2_O_2_ = 3:1) to eliminate any remaining organic residue. Subsequently, the wafer was immersed in a 5% HF solution to remove the native oxide layer and thorough rinsing with deionized (DI) water was performed to ensure a clean surface. To create an insulating layer, a thermal oxidation process was employed. The cleaned Si wafer was placed in a thermal chamber and subjected to a dry-wet-dry thermal oxidation process at a temperature of 1075 °C. This process resulted in the growth of a SiO_2_ layer with a thickness of 0.9 μm. After the oxidation process, the wafer was dried using N_2_ gas.

**Figure 7:**
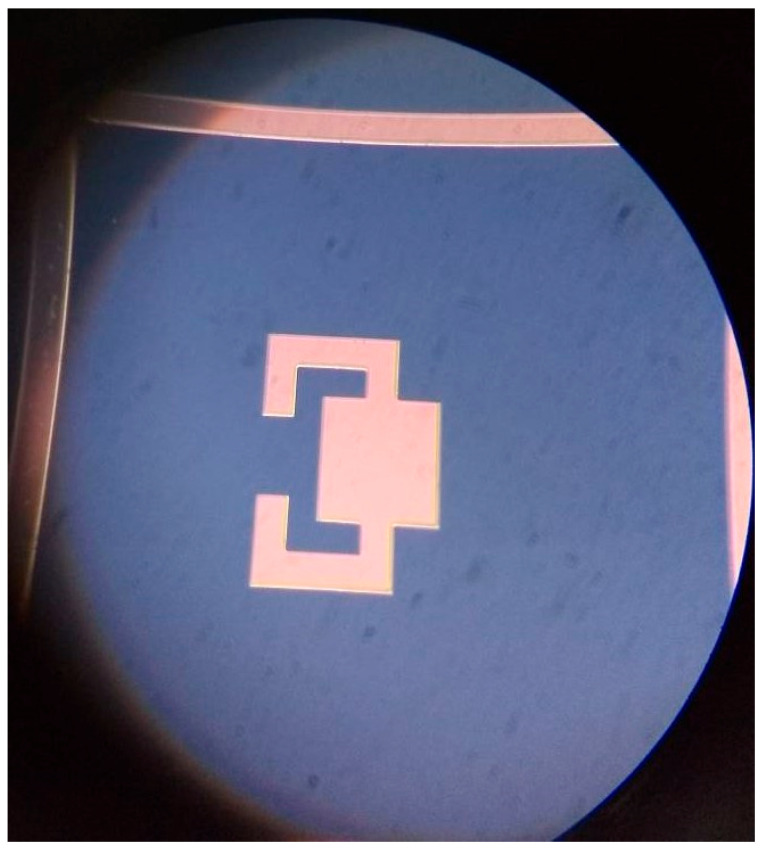
Microscopic view of the bottom electrode after the patterning.

The deposition of the bottom electrode was carried out using the e-beam evaporation technique. Aluminum (Al) was chosen as the material for the bottom electrode, and a layer with a thickness of 150 nm was deposited onto the SiO_2_ layer. The patterning of the bottom Al electrode was achieved using standard bright field photolithography, combined with the application of aluminum type-F chemical etchant. Figure 7 provides a microscopic view of the bottom electrode after the patterning process on the SiO_2_ layer. The image demonstrates the successful and precise patterning of the bottom Al electrode. The compatibility of the bottom SiO_2_ layer with the aluminum type-F chemical etchant is evident from the results, further confirming the effectiveness of the fabrication process.

These fabrication steps are crucial in establishing the foundation for the subsequent layers and components of the device. The careful cleaning and oxide growth processes ensure a clean and well-prepared surface, while the precise deposition and patterning of the bottom electrode contribute to the overall functionality and performance of the device.

The RF reactive magnetron sputtering system is used to grow the bulk zinc oxide layer on the bottom Al electrode at room temperature. This bulk ZnO layer was patterned with bright field photolithography and then etched using a wet chemical etchant (1% hydrochloric acid) solution. DC sputtering was used to deposit 20 nm Cr and a 130 nm thin Au layer as the top electrode of the resonator and patterned using a liftoff process.

**Figure 8:**
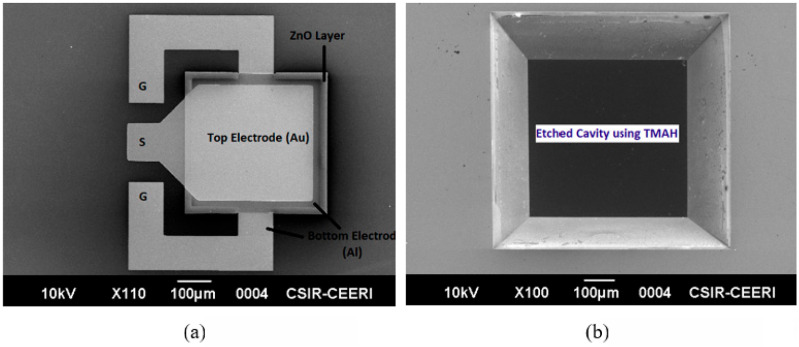
SEM diagram of the (**a**) top view of single-port cavity-based FBAR, and (**b**) slanted view of the backside cavity.

The dark field photolithography and buffered oxide etchant (BOE) were applied to pattern the backside of the SiO_2_ layer. Then, a TMAH solution was used for the etching of bulk Si to form the backside cavity. Figure 8a,b represent the SEM top view of the single-port cavity-based FBAR and a slanted view of the backside cavity, respectively. The active layer stack was successfully patterned because the surface exhibited a clean profile free of inhomogeneities.

## 4. Result and Discussion

### 4.1. Study of ZnO Morphology

**Figure 9:**
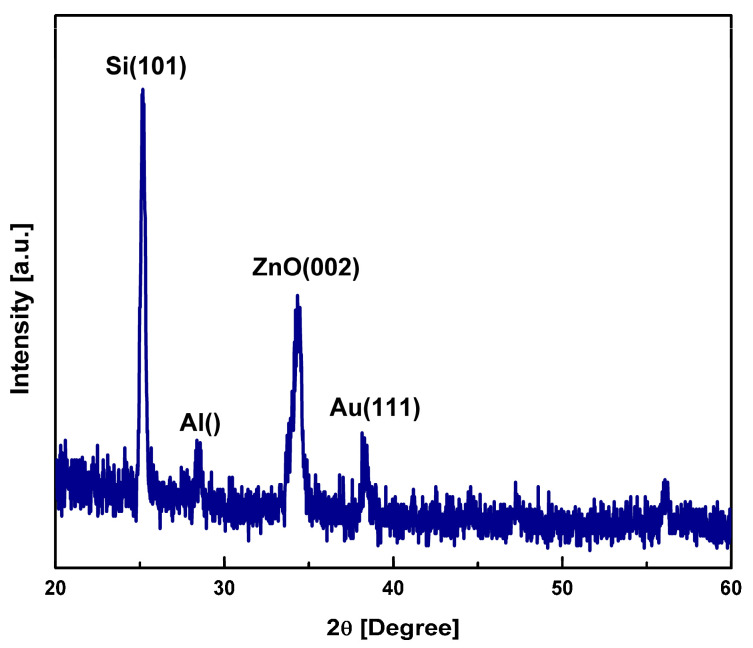
Grown ZnO layer, XRD pattern.

The crystalline phase of the deposited ZnO thin layer was determined using the PANalytical X’pert Powder system, equipped with a CuKα1 X-ray source with a line focus and a radiation wavelength of 1.54059 Å at 1.6 KW. Figure 9 displays the X-ray diffraction (XRD) pattern of the deposited piezoelectric ZnO film, scanned within the 2θ range of 20° to 60°. The XRD pattern reveals the presence of a highly crystalline film with a prominent (002) phase at 2θ = 34.58°, closely matching the reference peak indicated by JCPDS card no. 36-2828 [46]. Notably, the film exhibited negligible strain. The XRD analysis confirmed the excellent crystallinity and structural quality of the deposited ZnO thin film. The dominant (002) phase peak indicates the preferred orientation and alignment of the crystal lattice along the ZnO film growth direction. The observation of a single crystalline nature signifies the absence of significant defects or grain boundaries that could adversely affect the film performance.

The agreement between the experimental results and the reference peak from the JCPDS card further validates the accurate determination of the film’s crystallographic phase. This conformity provides confidence in the quality and reliability of the deposited ZnO thin film for its intended applications. The precise characterization of the crystalline phase is crucial in piezoelectric materials, as it directly influences their electromechanical properties. A well-defined and single crystalline structure ensures an improved piezoelectric response, high coupling coefficients, and enhanced performance in devices utilizing the ZnO thin film.

**Figure 10:**
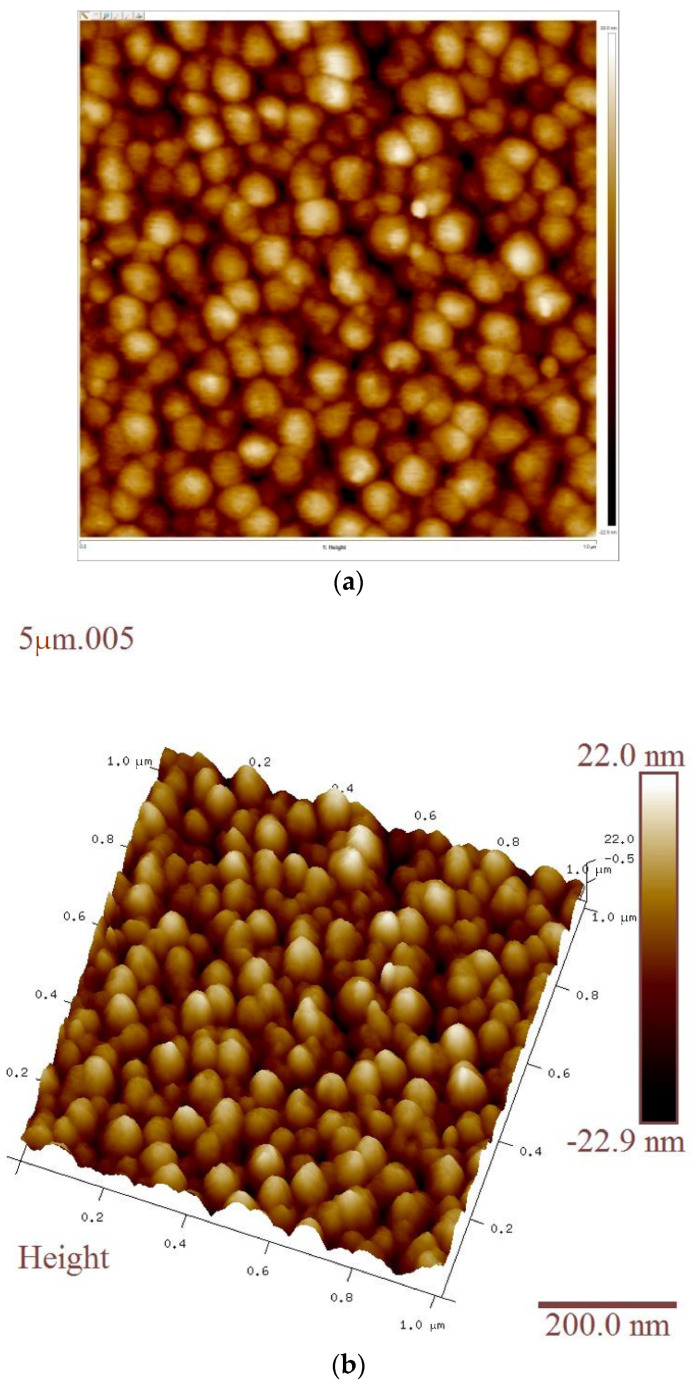
(**a**) Two-dimensional atomic force microscopy plot; (**b**) 3D atomic force microscopy image.

The morphology of the deposited ZnO film was inspected using atomic force microscopy (AFM, Brukar multimode 8). Figure 10a shows AFM micrographs which display that the ZnO film is uniformly distributed on the entire Si substrate and has a very low roughness of around 6.71 nm (calculated for 1 μm × 1 μm area). It is evident that the deposited film has a very smooth surface and a small grain size. The 3D micrograph in Figure 10b also depicts the uniform distribution of the grains in the ZnO film. The two-dimensional AFM micrograph represents the distribution of particles as well as the horizontal size of particles, something which cannot be achieved by a 3D AFM diagram.

**Figure 11:**
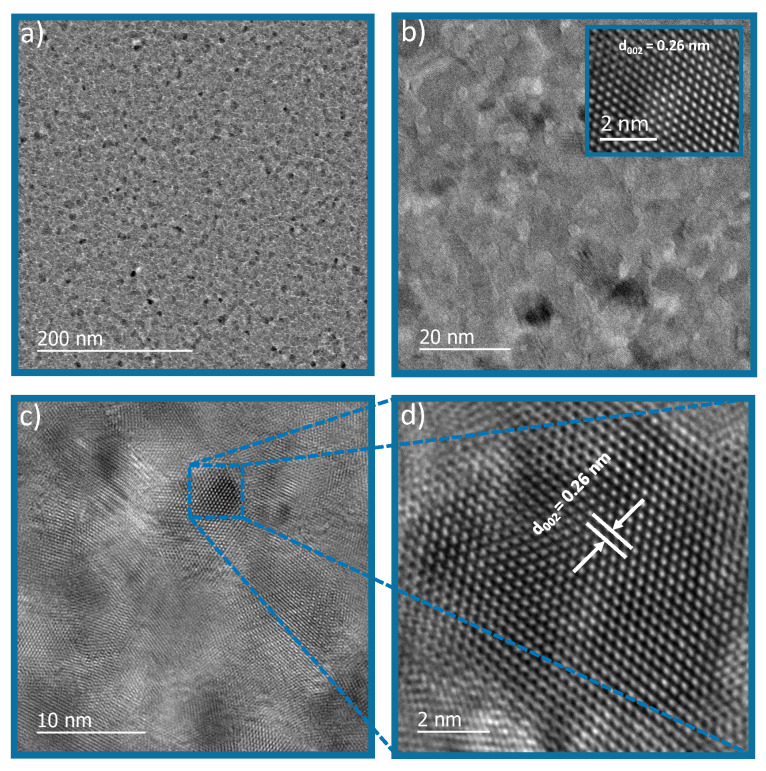
TEM micrograph.

The morphologies of the as-prepared samples were analyzed by (JEM2100F) transmission electron microscope (TEM) and high-resolution transmission electron microscope (HRTEM). TEM images in Figure 11 show an overview of the ZnO thin film showing nanorod-like morphology. The inset in Figure 11a shows the TEM micrograph, which shows that the film has grown uniformly throughout the substrate; there are no cracks in the film. Figure 11b,c show the rod morphology, with a rod diameter of 15 nm to 20 nm. Figure 11d and the inset in Figure 11b show the HRTEM image of ZnO nanorods aligned in 002 plane, a phenomenon which was also confirmed by XRD spectroscopy.

**Figure 12:**
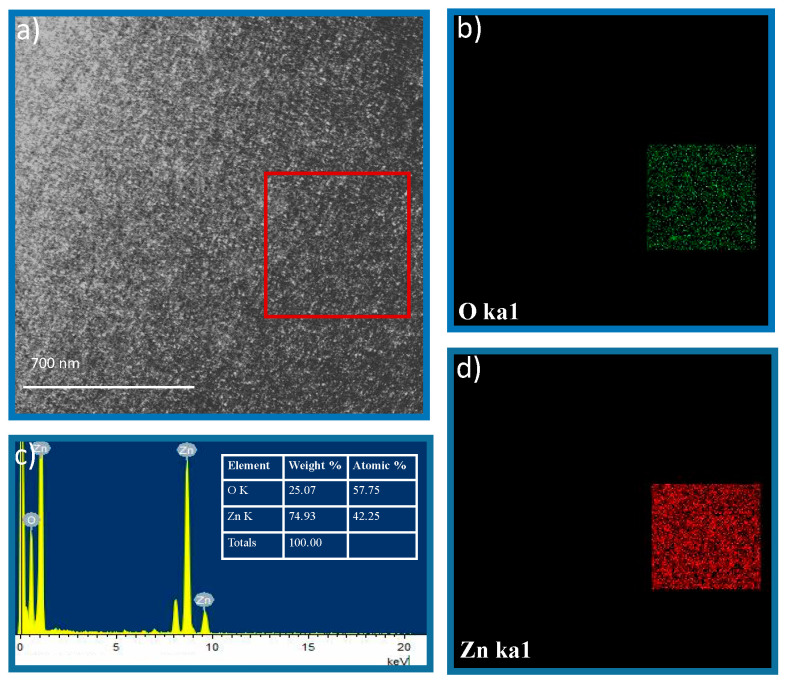
The elemental mapping of the TEM image and EDS spectra.

The elemental mapping of the as-prepared samples was analyzed by TEM. The spectra obtained from the EDS, shown in Figure 12c, confirm the qualitative elemental purity of the material. Quantitative EDS analysis confirmed the uniform distribution of Zn and O elements. The peak at 1.037 eV corresponds to the Lβ1 of Zn, 8.63886 corresponds to the Kα1 of Zn, 9.5720 corresponds to the Kβ1 of Zn, and 0.525 corresponds to the Kα1 of O.

The elemental homogeneity of the samples was checked using an EDS mapping technique. The elemental maps micrographs of Zn and O show a uniform density distribution, confirming the uniform distribution of Zn and O throughout the material. The obtained EDS maps are presented in Figure 12. Figure 12b,d show the elemental mapping of Zn and O, respectively, for the red rectangle visible in Figure 12a.

### 4.2. Radio Frequency Characteristic of the Resonator

**Figure 13:**
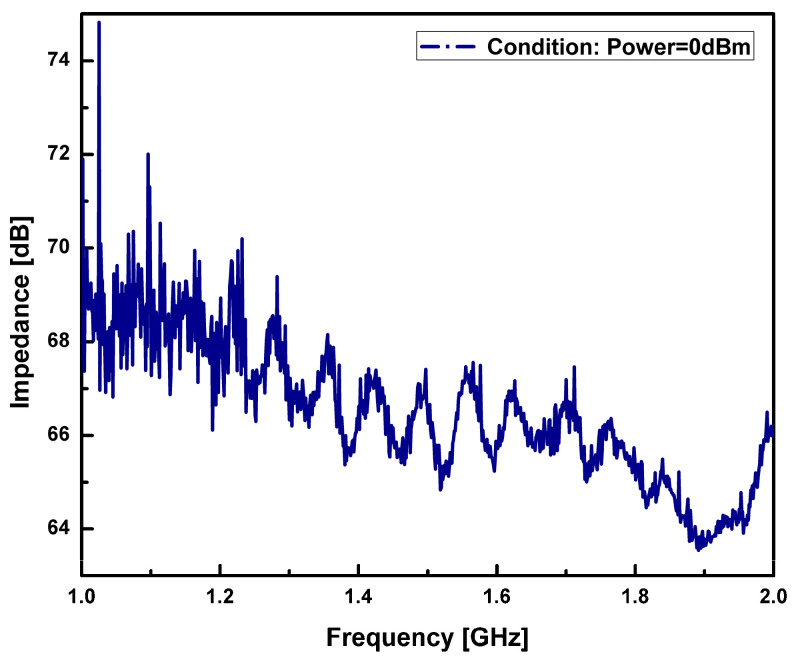
Measured impedance characteristic of a resonator without cavity.

The Radio Frequency characteristic of the single-port cavity-based FBAR was characterized with respect to impedance on an RF coplanar probe station using GSG-250 probes. A vector network analyzer (Model MS2028C, Anritsu, Hongkong) was used to measure the radio frequency response of the resonators from 1 GHz to 2.5 GHz. The fabricated device has an active area of 310 µm × 310 µm and a backside cavity whose dimensions are 300 µm × 300 µm. The resonator impedance, phase, effective electromechanical coefficient of coupling, and Q-factor parameters were plotted. Figure 13 shows the measured impedance (Z) and the phase (***ϕ***z) characteristic of the fabricated single-port resonator without the cavity as a function of frequency.

**Figure 14:**
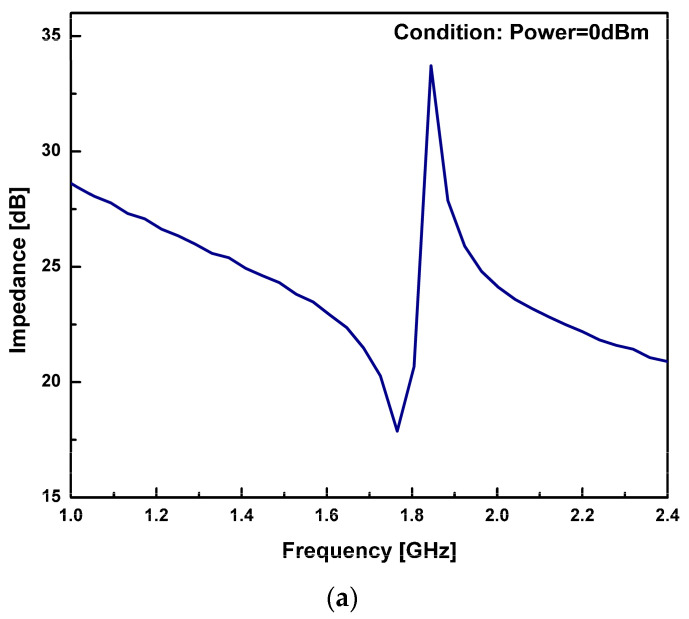

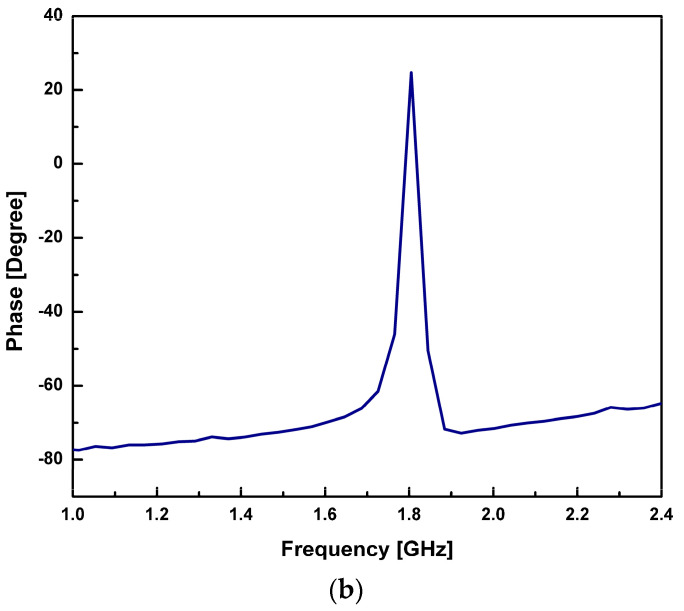
(**a**) Measured impedance characteristic of a cavity-based resonator; (**b**) measured phase characteristic for a cavity-based resonator.

The impedance (Z) and phase (***ϕ***z) characteristic of the fabricated single-port resonator with cavity are presented in Figure 14a,b, respectively. As it can be observed from Figure 14a, the resonant frequency, fr, had a value of 1.765 GHz and the anti-resonant frequency, fa, had a value of 1.844 GHz at a minimum impedance of 17.88 dB and at a maximum impedance of 33.71 dB, respectively, with $k_{eff}^{2}$ equivalent to 10.57%. As it can be seen, there was a perfect match between the resonant frequencies for the FBAR FEM simulation, with a minimal error of 5 MHz. The resonator’s quality factors Qr and Qa were estimated at 94.3 and 214, respectively, with corresponding Figure of Merit (FoM1) values of 9.97 and 22.6, respectively.

The finite-element-based simulation results were confirmed through practical RF measurements of the fabricated single-port cavity-based acoustic resonator. Table 1 shows the comparison of simulated and measured RF performance parameters of fabricated single port cavity based acoustic resonator. It is confirmed from the Table 1 that the confinement of acoustic signal has been improved by active area optimization technique. The measured performance parameter of the FBAR depends on various factors: the quality of ZnO, SiO_2_, BE, TE layer and bulk etching of Si using the TMAH process. This illustrates the reason for the gap between the simulation and fabrication results in Table 1. There is difference in the quality factor and coupling coefficient. This difference is due to the tradeoff between the coupling coefficient and quality factor. The value of coupling coefficient is increasing at the cost of reducing the quality factor [47,48]. By the selection of active area, the effects of higher harmonic modes, undesired coupling and spurious modes from the frequency response can be well suppressed.

The silicon bulk micromachining process simplifies fabrication of freely suspended membrane-based structures using wet chemical etching. However, the complete bulk etching of silicon substrate below the active area through wet chemical etching is a significant process and residuals of Si can cause the resonant and anti-resonant modes of the resonator to vanish. The effect of such residuals of Si substrate on resonant mode was observed. The study illustrates the generation of a resonant mode in bulk Si substrate with 280 µm thickness and completely etched Si substrate below the active area, shown in Figure 13 and Figure 14a.

Table 2, finally, compares the fabricated single-port cavity-based acoustic resonator results with those reported in the literature. The measured results are in agreement with those observed from the finite element analysis. This shows that the fabricated single-port cavity-based acoustic resonator gives better RF performance, thereby making it a promising device for sensing applications in future.

## 5. Conclusions

In conclusion, this manuscript has demonstrated the successful fabrication and opti-mization of a single port cavity based acoustic resonator utilizing zinc oxide as a piezoelectric layer. Through a comprehensive approach, the study harnessed finite element simulation, software-driven design, and advanced fabrication techniques to enhance the radiofrequency (RF) performance of the resonator. The design process incorporated finite element simulation within the COMSOL Multiphysics v4.4 software suite, enabling the refinement of the single port FBAR’s performance characteristics. Leveraging an opti-mized design parameter, the resonator’s mask was meticulously crafted using L-edit software, ensuring precise control over its structural aspects. The fabrication process itself underwent careful optimization, particularly in the deposition of the bulk ZnO layer on the bottom electrode. The emphasis on the (002) plane and the quality of the bulk ZnO layer was pivotal, as these factors significantly influence the confinement of the acoustic signal within the resonator’s active area and backside cavity. X-ray diffraction (XRD) and atomic force microscopy (AFM) analyses confirmed the exception-al single crystalline nature of the deposited zinc oxide film, marked by its dominant (002) plane orientation, small grain size, and remarkably smooth surface. Notably, the utilization of the TMAH jig for silicon bulk etching played a crucial role in safeguarding the integrity of the frontside active devices while optimizing the etch rate. The RF measurement phase involved the use of a Vector Network Analyzer (VNA), which offered insights into the impedance and phase spectra of the FBAR. The impedance response underscored the remarkable capabilities of the single port FBAR, notably in its effective suppression of spurious modes and higher harmonic modes, resulting in pristine confinement of the acoustic signal. The resonator’s key parameters were ascertained through comprehensive analysis. Its resonant frequency was found to be 1.765 GHz with a quality factor (Qr) of 94.3, while the anti-resonant frequency was measured at 1.844 GHz with a quality factor (Qa) of 214. The corresponding Figures of Merit (FOMs) for the resonant and anti-resonant frequencies were calculated to be 9.97 and 22.6, respectively. In essence, this research not only advanced the understanding of single port cavity based acoustic resonators but also showcased their immense potential as a platform for future gas-sensing applications. The successful integration of advanced simulation techniques, software-driven design, and meticulous fabrication processes has paved the way for enhanced RF performance and precise signal confinement. This work serves as the development of innovative and efficient gas sensing technologies, contributing to the further evolution of sensor-based applications.

## Figures and Tables

**Table 1:** Comparisons of simulated and measured RF performance parameters of proposed single-port cavity-based acoustic resonator.

S. No.	Parameter	Active Area of 225 × 225 µm^2^	Active Area of 310 × 310 µm^2^	Measured
1.	f_s_ [GHz]	1.762	1.762	1.765
2.	f_p_ [GHz]	1.802	1.82	1.844
3.	Q_s_	1046	906.6	94.3
4.	Q_p_	992	612.1	214
5.	$k_{eff}^{2}$ [%]	5.47	7.86	10.56

**Table 2:** RF performance parameters of the proposed single-port cavity-based acoustic resonator.

References	Device Name	PZE Material	f_r_ (GHz)	f_a_ (GHz)	$k_{eff}^{2}$	Q_s_	FoM_1_
[49]	FBAR	ZnO	1.546	1.590	6.83	350	23.9
[50]	FBAR	ZnO	1.498	1.513	2.39	224	5.35
[51]	FBAR	ZnO	1.43	1.441	1.88	433	8.14
[52]	FBAR	ZnO	2.11	2.127	2.03	253.2	5.14
This work	FBAR	ZnO	1.765	1.844	10.57	214	22.6

## Data Availability

The data presented in this study are available on request from the corresponding author.
